# Who seeks treatment for cannabis use? Registered characteristics and physical, psychological and psychosocial problem indicators among cannabis patients and matched controls

**DOI:** 10.1186/s12889-018-5625-0

**Published:** 2018-06-22

**Authors:** Solveig Glestad Christiansen, Anne Line Bretteville-Jensen

**Affiliations:** Norwegian Institute of Public Health, PO Box 222, Skoyen, N-0213 Oslo, Norway

**Keywords:** Cannabis, Substance Abuse Treatment, Norway

## Abstract

**Background:**

There has been an absolute and relative increase in the number of patients with cannabis-related disorders as the principal diagnosis in many countries in recent years. Cannabis is now the most frequently mentioned problem drug reported by new patients in Europe, and cannabis patients constituted one third of all drug treatment patients in 2015. There is limited knowledge with regard to patient characteristics, the extent and types of health and psychosocial problems, as well as their association with long-term outcomes.

**Methods:**

We analysed indicators of physical, psychological and psychosocial problems of all patients admitted to treatment for cannabis use in Norway in 2009 and 2010 using register data and observed them to the end of 2013. Patient characteristics and outcomes were compared to a randomly drawn control group with corresponding age and gender distribution. Using logistic regression of prospective data, we studied associations between baseline characteristics and work and study status in 2013.

**Results:**

Cannabis patients tended to be relatively young and the large majority were male. They had parents who were less highly educated compared to controls, while there was no difference in migration background. In addition to an increased risk of premature death, nearly half of the patients received a secondary psychological diagnosis and a similar proportion received an additional substance use diagnosis during the 4–5 years of study follow-up. The cannabis patients were less educated than the control group and also less likely to be studying or working at the end of the study period. Entering treatment at a young age, having completed more than secondary education, having a highly-educated mother and not having a secondary diagnosis were factors that were positively associated with being in education or employment at the end of follow-up.

**Conclusions:**

Data covering the entire Norwegian population of patients admitted primarily for cannabis-related problems showed comprehensive and complex patterns of physical, psychological and psychosocial problems. The prevalence and extent of these problems varied markedly from those of the general population. Work and study outcomes following treatment depended on the seriousness of the condition including co-morbidity as well as social capital.

## Background

The number of patients entering treatment with cannabis as their main problem drug has increased in absolute and relative terms in recent years. In Europe, cannabis is now the most frequently mentioned problem drug reported by new patients [1], and from 2006 to 2015 EU countries experienced a 77% rise in the number of first-time entrants for cannabis-related problems (from 43,000 to 76,000 patients) [1]. In 2015, cannabis clients constituted 32% of all drug treatment patients in the EU compared to 20% in 2006, and more than 132,000 patients were registered this year (http://www.emcdda.europa.eu/data/stats2017/tdi). Countries such as the USA and Australia have also experienced a significant increase in the number of cannabis patients [2–4] and nearly 3 out of 10 cannabis users in the USA manifested a cannabis-use disorder [2]. The high and increasing number of patients engender substantial costs to both individuals and society.

Most cannabis users, however, experience no, or very few, problems related to their drug use. Many only experiment with the drug a few times during adolescence or young adulthood, while a minority adopt a more regular and long-lasting consumption pattern. Studies suggest that roughly one in ten who have tried cannabis develop a dependency, and the number increases to one in six if cannabis use is initiated in adolescence [5]. Among daily cannabis users, 33%–50% may develop a dependency [6]. The risk of adverse consequences increases the younger a person starts using cannabis and the more frequently it is used, i.e. uptake in adolescence (before the brain is fully developed) and regular, long-term and high-dose use, increases the risk of developing physical and mental health problems, as well as psychosocial problems. Physical health problems may include respiratory diseases, cardiovascular diseases and cancer, while depression, anxiety and suicidal ideation constitute some of the mental health problems that have been linked to cannabis use [7]. Psychosocial difficulties can arise from factors such as dropping out of school, poor school and work performance and increased use of other substances [5, 7, 8].

Among the subgroup experiencing problems related to cannabis use, only a minority seek treatment. A major US study found that only 13% of those diagnosed with a cannabis disorder sought treatment [9] and other studies have reported that less than one third of all dependent cannabis users sought treatment during the past year [10, 11]. Nonetheless, the relatively high prevalence of frequent cannabis use in developed countries implies that even if only a small proportion of people seek treatment this will add up to a sizeable number of patients. There is a substantial diversity across countries and regions with regard to the types of treatment offered, the most common being outpatient visits to a physician, psychologist or some other professional, followed by inpatient treatment in a drug detoxification or rehabilitation unit, hospital ward or treatment in an emergency department [12]. Commonly, cannabis treatment relies on psychosocial approaches combining elements of individual and family therapy with social support and care.

Currently, there is limited knowledge with regard to patient characteristics, the extent and types of health and psychosocial problems as well as their association with long-term outcomes. Previous research suggests that males have a higher risk of developing cannabis dependence [13], and roughly 70% of cannabis patients are male. While more males than females use the drug frequently in the general population, the gender gap was even larger among those entering treatment [12]. Cannabis patients are younger than other patients with substance use disorders, and more than half of them are below the age of 30 [14]. Few report having a steady partner [10].

Many previous studies have relied on small samples from one or more treatment centres that may not have been representative of all cannabis patients and few have focused on the broad spectrum of cannabis-related problems. This study improves on this by using a national register covering all patients in Norway entering treatment with cannabis-related problems as their principal diagnosis in 2009 and 2010 and presents indicators of physical, mental and psychosocial problems. Although we cannot ascertain whether, or to what extent, these problems are caused by cannabis use, it is of interest to examine their prevalence in a population of cannabis patients. To study how these cannabis patients deviate from the general population with regard to personal characteristics and indicators of adverse consequences, we compare the patients to a control group matched on age and gender. As psychosocial problems among cannabis patients have been examined in previous studies to a lesser extent, we focus in particular on patients’ education and employment status. Further, by being able to link patient information to register data up to 2014, we investigate how individual factors and indicators of physical and mental health problems are associated with these psychosocial indicators at the end of the follow-up period.

## Methods

### Setting

Cannabis use, possession, production and sale remain illegal in Norway and the drug laws are *de facto* enforced. The lifetime prevalence of cannabis use of 15 and 16-year-old Norwegians is among the lowest in Europe (7% compared to an EU average of 17% in 2015, [15] and has decreased since 1999 [16]. About one in five adults (22%, 16–64 years) report ever having experimented with cannabis and 9% of young adults (16–34 years) report using the drug at some point during the last year [1].

The Norwegian healthcare system has universal coverage and is organised into primary and specialist healthcare sectors. Primary health care comprises services such as general practitioners (GPs) and low threshold treatment programmes, while specialist health care includes somatic and psychiatric hospitals and drug treatment centres. A referral from a GP or a limited number of social and health institutions is required to access specialist drug treatment. The Norwegian police and justice system, on the other hand, have no legal authority to refer users to specialist drug treatment. Once a referral is obtained, patients may freely choose from a certified list of public and private treatment centres and clinicians. The treatment is paid for by the state and is free at the point of delivery. The number of patients in specialist health care with cannabis as their main problem drug has increased with almost 40% in the period 2009-2014 [17].

### Study population

We employ data from the Norwegian Patient Registry (NPR), which covers the entire population of patients in the specialist healthcare system. The current sample encompasses all those who were admitted between 2009 and 2010 to treatment for substance use with cannabis-related disorders as the principal diagnosis, as diagnosed in accordance with the ICD-10 framework (*n*=3,951) [18]. The first admission in the period 2009–2010 will henceforth be referred to as the “index admission”.

We use prospective data on treatment received for cannabis use disorders and a range of other diagnoses from the index admission until the end of 2013. Using national ID numbers, the data from the NPR was linked to other national administrative registers containing data on demographic characteristics, causes of death, education and employment. The ID number is a unique 11-digit number used in all kinds of contact with the authorities such as applying for education, paying tax or registering at a new address, thus allowing individual-level data from different registers to be linked. To compare cannabis patients with the general population, a control group matched on age and gender was provided by Statistics Norway (*n*=7,902).

### Measures

For most variables, we used information registered in the year the patient was first admitted to treatment (2009 or 2010). Indicators for physical and mental health problems and psychosocial problems were chosen according to factors listed in the following recent reviews: “The health and social effects of non-medical cannabis use” [7] and “The Health Effects of Cannabis and Cannabinoids: The Current State of Evidence and Recommendations for Research” [8]. Being “in work” is defined as having been employed at some point during year t (either year of index admission or end of follow-up period). Likewise, being “in education” means having been enrolled in education in year t. All diseases were diagnosed and registered in accordance with the ICD-10 framework. Variables for secondary psychological diagnosis and substance use diagnosis (apart from cannabis) included all such diagnoses received from the time of the index admission to the end of the follow-up period.

### Analyses

Descriptive statistics of patients and controls are presented in Table 1 including p-values from t-tests and chi-square tests. Table 2 shows the characteristics of subgroups of patients defined by education and employment status at the end of the follow-up period. Unadjusted and adjusted odds ratios (OR) and 95% confidence intervals from bivariate and multivariate logistic models examining associations between baseline characteristics and being in work or education in 2013 are shown in Table 3. This table also shows logistic regression results for subgroups that changed the education or employment status (started or ended education or employment from baseline to end of follow-up, respectively).

**Table 1:** Demographics and problem indicators among cannabis patients and controls

		Cannabis patients (*n* = 3951)	Non-patients (*n* = 7902)	*p*-values
Personal characteristics				
Gender (%)	Male	74.6 (2947)	74.6 (5895)	
Age (%)	Less than 20 years	14.3 (565)	14.3 (2230)	
	20–24	32.4 (1280)	32.4 (2560)	
	25–29	23.6 (932)	23.6 (1865)	
	30–34	12.4 (490)	12.4 (980)	
	35–39	7.8 (308)	7.8 (616)	
	40 or older	9.6 (379)	9.6 (759)	
	*Mean age* (years)	27.2 (1075)	27.2 (2149)	
	*Range* (years)	15–67	15–67	
Marital status (%)	Married	5.3 (209)	19.3 (1525)	0.000
Background (%)	Norwegian	75.5 (2983)	73.6 (5816)	0.063
	Other western	15.4 (608)	16.6 (1311)	
	Non-western	9.1 (360)	9.5 (751)	
Father’s education (%)	Compulsory education	38.8 (1533)	26.4 (2086)	0.000
	Upper secondary school	46.3 (1829)	48.9 (3864)	
	Higher education	15 (593)	25.2 (1991)	
Mother’s education (%)	Compulsory education	45.1 (1781)	31.9 (2521)	0.000
	Upper secondary school	39.0 (1541)	43.2 (3414)	
	Higher education	15.9 (628)	24.9 (1968)	
Physical health problems				
Non-communicable	Chronic bronchitis	1.5 (59)	1.2 (95)	0.209
disease diagnoses (%)	Lung cancer	0.2 (79)	0.1 (79)	0.262
	Stroke	0.03 (12)	0	0.196
Mental health problems				
Psychological disease	Mood disorders	21.5 (849)	3.5 (279)	0.000
diagnoses (%)	Personality disorders	10.9 (431)	1.0 (79)	0.000
	Attention deficit disorders	13.1 (518)	1.0 (77)	0.000
	Neurotic disorders	21.9 (865)	3.5 (273)	0.000
	Schizophrenia	7.2 (284)	0.5 (37)	0.000
	*Any psychological diagnosis*	48.6 (1920)	6.8 (535)	0.000
Substance use	Alcohol	19.8 (782)	0.8 (64)	0.000
diagnoses (%)	Opioids	13.2 (522)	0.1 (9)	0.000
	Sedatives	18.6 (735)	0.1 (11)	0.000
	Cocaine	4.0 (158)	0.1 (7)	0.000
	Poly-drug use	27.4 (1083)	0.4 (35)	0.000
	*Any substance use diagnosis*	49.2 (1943)	1.2 (98)	0.000
Psychosocial indicators				
Completed education (%)	Compulsory education	73.1 (2888)	32.5 (2564)	0.000
	High school	809	40.1 (3169)	
	Higher education	3.67 (145)	22.8 (1798)	
	Missing	2.8 (109)	4.7 (371)	
	In education_admission_	16.5 (652)	29.8 (2355)	0.000
	In education_2013_	11.7 (462)	18.3 (1446)	
	In work_admission_	32.9 (1300)	73.9 (5840)	0.000
	In work_2013_	35.4 (1399)	78.6 (6211)	
	In educ. or work_admission_	43.4 (1715)	84.3 (6661)	
	In educ. or work_2013_	41.6 (1644)	83.4 (6590)

**Table 2:** Demographics and problem indicators among cannabis patients across education and work status subgroups at end of follow-up

		Not in education or work (*n* = 2309)	In work or education (*n*=1642)	In work (*n*=1397)	In education (*n*=462)
Personal characteristics					
Gender (%)	Female	24.2^a^ (559)	27.1 (445)	26.2 (366)	32.2 (149)
Age (%)	Less than 20 years	29.9 ^a^ (690)	36.0 (591)	36.8 (514)	36.6 (169)
	20–24	22.7 ^a^ (524)	24.8 (407)	24.0 (335)	23.4 (108)
	25–29	13.2 ^a^ (305)	11.3 (186)	11.4 (159)	8.9 (41)
	30–34	9.2 ^a^ (212)	5.7 (94)	5.9 (82)	3.2 (15)
	35–39	12.7 ^a^ (293)	5.2 (85)	5.9 (82)	1.8 (8)
	40 or older	12.3 ^a^ (284)	17.0 (279)	16.0 (224)	26.1 (121)
	*Mean age* (years)	28.3 (653)	25.6 (420)	25.8 (360)	23.6 (109)
	*Range* (years)	15-67	15-61	15-61	15-50
Marital status (%)	Married	5.3 (122)	5.2 (85)	5.9 (82)	1.5 (7)
Background (%)	Norwegian	76.7 (1771)	73.9 (1213)	74.6 (1042)	70.4 (325)
	Other western	14.2 (328)	17.0 (279)	16.3 (228)	19.7 (91)
	Non-western	9.1 (210)	9.1 (149)	9.1 (127)	9.9 (46)
Social Background					
Father’s education (%)	Compulsory education	42.2 ^a^ (974)	34.0 (558)	35.3 (493)	39.4 (182)
	Upper secondary school	45.0 ^a^ (1039)	48.1 (790)	48.2 (673)	43.9 (203)
	Higher education	12.8 ^a^ (296)	17.9 (294)	16.5 (231)	16.7 (77)
Mother’s education (%)	Compulsory education	40.7 ^a^ (925)	38.6 (634)	40.2 (562)	30.3 (140)
	Upper secondary school	47.4 ^a^ (1094)	40.0 (656)	40.3 (563)	38.0 (176)
	Higher education	11.9 ^a^ (275)	21.4 (351)	19.5 (272)	31.7 (146)
Mental health problems					
Psychological diagnoses (%)	Mood disorders	23.0 ^a^ (531)	19.4 (319)	18.5 (258)	22.3 (103)
	Personality disorders	13.8 ^a^ (319)	6.9 (113)	6.7 (94)	7.6 (35)
	Attention deficit disorders	15.7 ^a^ (363)	9.5 (155)	9.8 (137)	7.1 (34)
	Neurotic disorders	24.9 ^a^ (575)	17.8 (292)	16.6 (232)	19.3 (89)
	Schizophrenia	9.4 ^a^ (217)	4.0 (66)	3.4 (47)	7.4 (34)
	*Any psychological diagnosis*	53.8 ^a^ (1242)	41.2 (677)	37.2 (520)	40.5 (187)
Substance use diagnoses (%)	Alcohol	22.0 ^a^ (508)	16.7 (274)	16.1 (225)	16.5 (76)
	Opioids	17.9 ^a^ (413)	6.6 (108)	5.8 (81)	8.0 (37)
	Sedatives	23.7 ^a^ (547)	11.5 (189)	10.5 (147)	13.6 (63)
	Cocaine	3.4 ^a^ (79)	4.9 (80)	5.2 (73)	3.5 (16)
	Poly-drug use	33.4 ^a^ (771)	18.9 (310)	17.4 (243)	23.2 (107)
	*Any substance use diagnosis*	56.8 ^a^ (1312)	38.4 (631)	37.2 (520)	40.5 (187)
Psychosocial indicators					
Completed education (%)	Compulsory education	77.4 ^a^ (1786)	67.1 (1102)	67.4 (941)	65.6 (303)
	Upper secondary school	16.6 ^a^ (384)	25.9 (425)	25.2 (353)	27.3 (126)
	Higher education	3.4 (78)	5.1 (84)	5.2 (73)	5.8 (27)
	Missing	2.6 (61)	1.9 (31)	2.2 (30)	1.3 (6)

^a^difference between those not in education or work (column 1) and those in education or work (column 2) significant at the 0.05 level

**Table 3:** Status and changes in status of employment and education at the end of follow-up. Logistic regression, odds ratios (OR), (95% CI)

		In education or work (*n*=1642) Unadj. OR	In education or work (*n*=1642) Adj. OR	Started studying or working (*n*=611)	No longer studying or working (*n*=684)
Personal characteristics					
Gender	Female	1.16 (1.00–1.34)	1.15 (0.98–1.34)	1.01 (0.80–1.27)	0.76 (0.60–0.96)
Age	Less than 20 years	1.28 (1.03–1.57)	1.48 (1.18–1.85)	1.13 (0.80–1.59)	0.77 (0.55–1.07)
	20–24	1.11 (0.93–1.31)	1.24 (1.04–1.49)	1.10 (0.86–1.42)	0.82 (0.62–1.08)
	25–29	1	1	1	1
	30–34	0.79 (0.63–0.98)	0.71 (0.56–0.89)	0.57 (0.41–0.80)	0.93 (0.64–1.35)
	35–39	0.57 (0.43–0.75)	0.57 (0.42–0.75)	0.54 (0.37–0.80)	1.29 (0.80–2.07)
	40 or older	0.37 (0.28–0.49)	0.37 (0.28–0.50)	0.25 (0.16–0.39)	1.06 (0.66–1.69)
Country background	Norwegian	1	1	1	1
	Other western	1.24 (1.04–1.47)	1.16 (0.95–1.40)	1.03 (0.78–1.37)	0.80 (0.60–1.07)
	Non-western	1.04 (0.83–1.30)	1.76 (0.83–1.38)	0.93 (0.64–1.37)	0.80 (0.55–1.17)
Social Background					
Father’s education	Compulsory edu.	1	1	1	1
	Upper secondary school	1.30 (1.12–1.51)	1.16 (0.99–1.35)	0.94 (0.75–1.17)	0.78 (0.61–0.99)
	Higher education	1.71 (1.40–2.09)	1.16 (0.92–1.46)	1.06 (0.76 –1.49)	0.92 (0.66 –1.29)
Mother’s education	Compulsory edu.	1	1	1	1
	Upper secondary school	1.34 (1.16–1.55)	1.23 (1.05–1.43)	1.22 (0.98–1.52)	0.83(0.65–1.05)
	Higher education	2.30 (1.90–2.78)	1.76 (1.42–2.18)	1.75 (1.27–2.41)	0.61 (0.44–0.83)
Mental health problems					
	*Any psychological diagnosis*	0.60 (0.53–0.68)	0.56 (0.50–0.66)	0.63 (0.52–0.77)	1.89 (1.54–2.33)
	*Any substance use diagnosis*	0.47 (0.42–0.54)	0.53 (0.52–0.91)	0.62 (0.51–0.75)	1.86 (1.51–2.28)
Psychosocial indicator					
Completed education	Compulsory educ.	1	1	1	1
	Upper secondary	1.79 (1-53-2.10)	2.14 (1.80-2.56)	1.63 (1.24-2.15)	0.54 (0.42-0.70)
	Higher education	2.23 (1-59-3.13)	3.26 (2.22-4.78)	1.17 (0.56-2.44)	0.28 (0.16-0.50)

## Results

### Demographics and problem indicators among patients and controls

A large majority (74.6%) of cannabis patients were males; Table 1. The mean age at the time of index admission was 27.2 years, with an age range from 15 to 67 years. About 70% were 29 years of age or younger. Only 5% of patients were married compared to 19% of controls, while there was no statistically significant difference in migration background.

Social background, measured by parents’ education, however, differed markedly. We found that nearly 40% of cannabis patients had fathers who had only completed compulsory education compared to 26% among members of the control group. Similarly, 45% of patients and 32% of non-patients had mothers who had only completed compulsory education. A smaller proportion of patients had fathers (15.0%) or mothers (15.9%) who had completed higher education than in the control group (25.2% and 24.9%, respectively).

Apart from the risk of premature mortality, there was little difference between patients and controls regarding the indicators for *physical health problems*. The prevalence of chronic bronchitis and lung cancer was low and almost identical, while strokes were only registered for 12 patients and no one in the control group.

*Mental health problems* were significantly more prevalent among cannabis patients than in the general population (46% vs 7%). More than 20% were diagnosed with a mood disorder, 11% with a personality disorder, 13% with an attention deficit disorder, 22% with a neurotic disorder and 7% with schizophrenia during the study period. Further, nearly half of the patients received a diagnosis for an additional substance abuse; 20% for alcohol, 19% for sedatives and 13% for opioids. The same applied to only 1% of the control group.

Furthermore, *psychosocial* indicators, represented by education and employment status, differed substantially between cannabis patients and their controls. Almost three out of four cannabis patients (73.1%) had only completed compulsory education compared to one out of three (33%) of non-patients. A much smaller proportion of cannabis patients (16.5%) than non-patients (29.8%) were enrolled in education in the year of study inclusion, while 11.7% of patients compared to 18.3% of controls studied at the end of the follow-up period. Employment differed even more; just above 33% of patients were employed at baseline compared to nearly 74% of non-patients. More than twice as many controls than patients were employed at the follow-up (79% versus 35%, respectively). Roughly two out of five cannabis patients were either studying or working both at the time of inclusion and in 2013.

We found few systematic differences between male and female patients, except that female patients tended to be somewhat younger and a larger proportion of female patients had an additional psychological diagnosis (results not shown in the tables).

### Demographics and problem indicators among cannabis patients across education and work status subgroups at the end of the follow-up

Compared to patients who were neither studying nor working in the final follow-up year (2013), patients in employment or in education tended to be younger: Table 2 columns 1 and 2. A larger proportion of patients with a western background other than Norwegian was found among those who were studying compared to those who were not working or studying. Patients who were studying or working in 2013 also tended to have more highly educated parents. For example, 42% of those who were not studying or working had fathers who had only completed compulsory education compared to only 34% of fathers of patients who were working or studying. Similarly, 21% of patients who were working or studying had mothers with a higher level of education compared to only 12% of patients who were not working or studying in the final year. In particular, those who were studying had highly educated mothers (nearly 32%).

A larger proportion among those who were not working or studying had a secondary psychological diagnosis (54%) than among those who were working or studying (41%). There was also a higher proportion of patients with an additional substance use diagnosis among those who were not working or studying (57%) than among those who were students or employees (38%) and a smaller proportion had completed upper secondary school education (17% compared to 26%) at baseline.

### Predictors for final year outcomes

#### Univariate analyses of being in education or work at the end of follow-up

Being in education or work in 2013 was positively correlated with being female, being under 20 years of age and having a western background that was other than Norwegian: Table 3, column 1. Being in education was also positively associated with having parents who had completed upper secondary school or higher education and had completed more than compulsory education. Furthermore, studying or working in the final year of the follow-up period was negatively associated with having a secondary psychological or substance use diagnosis.

### Multivariate analyses

Controlling for baseline variables, we found that studying or working in the final year was positively associated with being less than 25 years of age, having a mother who had completed higher education as well as having completed upper secondary school or higher education: Table 3, column 2. In addition, studying or working was negatively associated with being older than 30 years of age and having a secondary psychological or substance use diagnosis.

Furthermore, we conducted a separate analysis of those who had started working or studying during the follow-up, i.e. those who were studying and working in 2013 but who were not studying or working at baseline: Table 3, column 3. When controlling for other variables, starting work or studying was positively associated with having a mother who had completed higher education and completed upper secondary school education. Starting working or studying was negatively associated with being older than 30 years of age and having been diagnosed with a secondary psychological or substance use disorder.

In the final analysis presented in column 4 in Table 3, we included only those patients who were working or studying at baseline but not at the end of follow-up. Controlling for all other variables, we found that no longer studying or working was negatively associated with being female, having a father who had graduated from upper secondary school, a mother who had completed upper secondary school or higher education. It was also positively associated with having a secondary psychological or substance use diagnosis.

## Discussion

The absolute and relative increase in the number of patients reporting cannabis as their principal problem drug is striking, not least because this rise is not reflected in the trend in cannabis use, which has levelled off during the same period [12]. This might partially be explained by an increased awareness about cannabis-related problems or by an increased acceptance of seeking help. Moreover, it has been suggested that recent changes in cannabis control and the regulatory framework, as well as changes in production, distribution and cannabis products have led to riskier and more intensive user patterns, thus resulting in a greater demand for treatment [1]. Furthermore, changes in risk perception, availability of more potent types of cannabis, treatment referral practices and levels of treatment provision may also have contributed to the observed increase. The number of patients in specialist health care with cannabis as their main problem drug has increased with almost 40% in the period 2009-2014 [17].

However, regardless of what may explain the upsurge in patients, cannabis use now represents a major issue for drug treatment services and constitutes an area of growing importance. Improved knowledge of patient characteristics and cannabis-related problems is warrented. In particular, there seems to be a knowledge gap with regard to psychosocial problems facing cannabis patients. In addition to studying patient characteristics relative to a control group, this study has provided detailed analyses of subgroups of cannabis patients defined by education and employment status. Furthermore, data linkage across registers has made it possible to examine long-term outcomes.

Our findings demonstrate that cannabis patients differed markedly from the general population with a corresponding age and gender distribution for most characteristics and problem indicators. In line with previous studies of cannabis patients, we found males to be largely overrepresented [19–21]. Although more males than females reported having ever used cannabis in Norwegian population surveys [1], this gender difference was not as extreme as the gender difference observed among those admitted to treatment. Previous research has not found significant gender differences in the probability of seeking treatment among those who have problems related to cannabis use [9, 13, 22]. It is therefore likely that the observed gender disparity is caused by males more often being heavy and frequent users and being at greater risk of developing cannabis addiction [23, 24].

Our patient population is somewhat older than what has been found in previous studies [19, 25]. As previously mentioned, the Norwegian legal system cannot refer cannabis users for treatment. In countries where cannabis patients can be referred by the police and the justice system, cannabis patients may well be younger. In both of the aforementioned studies, a considerable proportion of patients have been referred to treatment by the police or legal system.

Few cannabis patients were married (5%), a substantially lower proportion than found among the controls (19%). This might be explained by previous findings of cannabis users reporting lower levels of relationship satisfaction [26] and that cannabis use leads to lower perceived levels of interpersonal skills [27]. Furthermore, using cannabis has been linked to delayed marriage, as well as divorce [28].

Interestingly, we found an almost identical distribution of immigrant backgrounds (Norwegian, other western and non-western) among the cannabis patients as in the control group. Whether this reflects similar cannabis consumption patterns and a propensity to seek treatment among groups of different backgrounds is not known. Some previous studies have found lower levels of substance use among immigrants [29–31], while others reported higher levels of cannabis use among immigrant than non-immigrant adolescents [32, 33].

A much larger proportion of patients’ parents had only completed compulsory education compared to the general population. This is in line with previous findings suggesting that although adolescents from higher socioeconomic backgrounds are more likely to experiment with cannabis than those from somewhat poorer backgrounds, they are less likely to transition to frequent or heavy use [24, 34]. In addition, those from poorer backgrounds seem more likely to become dependent shortly after the onset of use [35]. Various *physical health problems* have been linked to frequent and/or long-term cannabis use. Given the fact that most users smoke the drug, a heightened risk of respiratory diseases, such as worsened lung function, asthma, or chronic bronchitis has been suggested, as well as an increased risk of certain types of cancer and some cardiovascular diseases [7, 8]. We did not find support for the claims in our data, as the mentioned diseases were low prevalent and of roughly similar magnitude among patients and controls. However, as these diseases may appear after long and intensive cannabis use, it should be noted that our follow-up period was relatively short and the cohort of cannabis patients relatively young.

Further, an increase in all-cause mortality has also been associated with frequent cannabis use [36, 37]. In our study, 2.5 percent of the patients died during the follow-up period (none in the control group), which is in line with what has been reported elsewhere [38].

Previous studies have further shown a high degree of comorbidity in cannabis patients, both in terms of *mental health problems* and *secondary substance abuse* [10, 19–21, 25, 39]. In line with this, we found that mental health problems varied substantially between cannabis patients and controls. Levels of psychological diagnoses and additional substance use diagnoses were high and much higher than in the general population. A positive relationship between a number of psychological diagnoses, such as depression [40], social anxiety [41], attention deficit disorder [42] and cannabis use has been established. Moreover, the high level of secondary substance use diagnoses is in accordance with earlier studies of cannabis patients [19, 20, 25]. This underlines that cannabis patients have comprehensive and complex health problems.

Our data suggest that cannabis patients suffer from psychosocial problems, here indicated by their relatively low level of education and their lower likelihood of studying and working. Both acute and long-term cannabis use, in particular during adolescence, has been related to impairments in subsequent academic achievement and education, employment and income, as well as social relationships and social roles [8, 10, 23, 43]. Most cannabis users started using the drug while they were in their teens [14, 44]. It is stated that the brain is often not fully developed before the age of 25 [45], which has lent support to the claim that adolescent cannabis use could negatively influence users’ memory, learning, and attention span [46, 47]. At the time of admission, a vast majority (73%) had completed no more than compulsory education, which is much higher than in the general population (33%). The proportion of the patients who were enrolled in education (16.5%) was also considerably lower than among non-patients (30%). Early cannabis users have been found to have poor school performance, early school leaving and leaving without qualifications [48–51]. In the year of treatment admission, employment was also much lower among patients than controls: 33% versus almost 74%.

Altogether, only 43.4% of patients were studying or working at the start of the study, i.e. a large proportion of Norwegian cannabis patients were so-called NEETs (not in education, employment or training). This is in line with previous research, which has found that cannabis users over the age of 20 are often marginalised individuals who are poorly educated and unemployed [52]. Regular cannabis use has been linked to reduced work commitment [53] and an increased risk of becoming unemployed [54, 55]. The NEET group was characterised as being male, having a higher than average age, being native Norwegians, having more health and mental problems and less formal education than patients who were studying or employed at the end of the follow-up.

The logistic regression results presented in Table 3 show how the probability of studying or working at the end of the follow-up period was associated with a range of personal characteristics, background variables and problem indicators. Not surprisingly, age and problem indicators, such as psychological or substance use co-morbidity, were negatively associated with working or studying during the final year. Females seemed to have a somewhat greater probability of positive final year outcomes although this was only significant if they were already working or studying at baseline. Having completed upper secondary school or higher education was also associated with better outcomes. It could be that the more highly educated are better at finding the right kind of treatment, and to comply with the treatment instructions given by health professionals. Employers might also be more accommodating regarding employees with previous substance use problems in jobs demanding a higher level of education.

Previous research has found that parental social support is a predictor of positive treatment outcomes [56]. In line with this, we found that having a mother who had completed higher education was associated with being in work or studying in the final year of the follow-up period. Those with more highly educated parents might feel under more pressure to continue studying beyond their compulsory education. It is also plausible that more educated mothers are better at gathering information about the types of treatment available and demanding the right type of treatment for their children, as well as giving their children more support.

Moreover, having mothers with a higher level of education was positively associated with starting work or studying during follow-up and negatively associated with no longer studying or working when the opposite was true at baseline. Magnitudes of associations for *starting* were almost identical to the results for *being* in education and employment at the end of the follow-up. Being female, mothers’ education and own educational attainment were negatively associated with no longer studying or working, while mental health problems, as expected, were positively associated with no longer studying or working.

### Strengths and limitations

Our data cover the entire population of Norwegians cannabis patients admitted to specialist treatment during 2009 and 2010. This is an improvement on many previous studies, which have mainly relied on data from one or more treatment centres. The profile of their patients may therefore not be representative of cannabis patients in the total population. In addition, having information from official registers meant that we did not encounter problems of non-response, attrition or recall bias. However, the registers do not provide information on the patient’s age at the onset of cannabis use or indications of the severity of the current problem. In addition, information on substance use treatment was only included in the Norwegian Patient Register in 2009, which means that we have no information on whether the index admission was the first-time treatment for cannabis-related problems.

## Conclusion

Data covering the entire Norwegian population of patients admitted primarily for cannabis-related problems from 2009 to 2010 showed that cannabis patients have comprehensive and complex patterns of physical, psychological and psychosocial problems. The prevalence and extent of these problems varied markedly from those of the general population. In addition to an increased risk of premature death, we found that nearly half of the patients received a psychological secondary diagnosis and a similar proportion received an additional substance use diagnosis during the 4–5 years of study follow-up. The cannabis patients were less educated than the control group and less likely to be in education or employment, including at the end of the study period. Entering treatment at a young age, completing more than secondary education, having a highly educated mother and not having a secondary diagnosis were factors positively associated with studying or working 4 to 5 years subsequent to admission.
